# Challenges of Research on Person-Centered Care in General Practice: A Scoping Review

**DOI:** 10.3389/fmed.2021.669491

**Published:** 2021-06-24

**Authors:** Jako S. Burgers, Trudy van der Weijden, Erik W. M. A. Bischoff

**Affiliations:** ^1^Dutch College of General Practitioners, Utrecht, Netherlands; ^2^Department of General Practice, Care and Public Health Research Institute, Maastricht University, Maastricht, Netherlands; ^3^Department of Primary and Community Care, Radboud University Medical Center, Nijmegen, Netherlands

**Keywords:** general practice, family practice, patient-centered care, patient outcome assessment, systematic review, review, personalized medicine

## Abstract

**Background:** Delivering person-centered care is one of the core values in general practice. Due to the complexity and multifaceted character of person-centered care, the effects of person-centered care cannot be easily underpinned with robust scientific evidence. In this scoping review we provide an overview of research on effects of person-centered care, exploring the concepts and definitions used, the type of interventions studied, the selected outcome measures, and its strengths and limitations.

**Methods:** Systematic reviews on person-centered care compared to usual care were included from Pubmed, Embase, and PsycINFO. The search was conducted in February 2021. Data selection and charting was done by two reviewers.

**Results:** The literature search yielded 481 articles. A total of 21 full-text articles were assessed for eligibility for inclusion. Four systematic reviews, published between 2012 and 2018, were finally included in this review. All reviews used different definitions and models and classified the interventions differently. The explicit distinction between interventions for providers and patients was made in two systematic reviews. The classification of outcomes also showed large differences, except patient satisfaction that was shared. All reviews described the results narratively. One review also pooled the results on some outcome measures. Most studies included in the reviews showed positive effects, in particular on process outcomes. Mixed results were found on patient satisfaction and clinical or health outcomes. All review authors acknowledged limitations due to lack of uniform definitions, and heterogeneity of interventions and outcomes measures.

**Discussion:** Person-centered care is a concept that seems obvious and understandable in real life but is complex to operationalize in research. This scoping review reinforces the need to use mixed qualitative and quantitative methods in general practice research. For spreading and scaling up person-centered care, an implementation or complexity science approach could be used. Research could be personalized by defining therapeutic goals, interventions, and outcome variables based on individual preferences, goals, and values and not only on clinical and biological characteristics. Observational data and patient satisfaction surveys could be used to support quality improvement. Integrating research, education, and practice could strengthen the profession, building on the fundament of shared core values.

## Introduction

Delivering person-centered care is one of the characteristics of general practice and part of the competencies of the general practitioner (1). In many European countries person-centered care is also considered as core value in family medicine (2). In a recent survey among Dutch general practitioners and trainees, person-centered care had the highest score from a set of 19 potential values (3).

Despite broad consensus on the importance of person-centered care in general practice, a common definition is lacking (4). In literature, for instance, person-centered care and patient-centered care are often considered as similar concepts but differ in its goals (5, 6). Person-centered care broadens the perspective of patient-centered care by aiming to live a meaningful life beyond functional well-being. Clarifying the concepts is necessary for interpretation and use of research findings in general practice (7).

Research into person-centered care can include different interventions, such as training for providers in communication and management and promotion of patient empowerment and engagement (8, 9). Measuring effects of person-centered care needs a clear view on outcomes of interest and selection of validated instruments (10).

In contrast to the strong belief in the need for person-centered care in primary care, the evidence on efficacy is limited due to poor designs or lack of methodological quality (11). In this review we aim to provide an overview of research studying the effects of person-centered care on patient outcomes in general practice, including strengths and limitations. Specific objectives are to explore: (i) the concepts and definitions used; (ii) the type of interventions studied; and (iii) the selected outcome measures. The information gained from this study could contribute to improving design and quality of future studies on person-centered care in general practice.

## Methods

We conducted a scoping review as we were interested in how research is conducted on this topic and to identify factors related to the concept of person-centered care (12). The quality of evidence is not evaluated in a scoping review in contrast to systematic reviews. For this scoping review, we adopted the methodological framework developed by Arksey and O'Malley (13).

### Identifying Relevant Studies

A pilot literature search in Pubmed using MeSH terms “Patient-Centered Care,” “Primary Health Care,” “General Practice,” “Patient Outcome Assessment,” and “Quality of Life” resulted in 140 randomized controlled trials and 40 systematic reviews. As we did not aim for completeness, the final search was limited to systematic reviews. Medline, Embase, and PsycInfo databases were searched on February 1st, 2021 with no language or date restriction. The search strategy is included in Appendix 1 in Supplementary Material.

### Selecting Studies

To be eligible for inclusion, the article had to review studies to the effects of interventions promoting person -centered care in primary care, including randomized trials comparing an intervention group with usual care. In addition, patient relevant outcomes had to be measured using validated instruments. Protocols of trials were excluded. Articles were also excluded when they focused on: (a) introduction of specific decision aids or other tools for patients; (b) one specific disease; (c) management and coordination of care; (d) hospital care.

After screening title and abstract of each citation, the articles eligible were assessed by two reviewers. Final selection of articles was based on consensus.

### Charting the Data

A data-charting form was developed to determine which variables to extract. The form contained descriptive variables (year of publication, number of studies included in the systematic review, setting, and country) and information about the way authors defined person-centered (or patient-centered) care, type of interventions studied, reported outcomes, and the effects of the interventions. The data were described according to the authors of the review. The strengths and limitations of the studies included in the reviews were extracted from the discussion sections of the articles.

### Collating, Summarizing, and Reporting the Results

As we were particularly interested in the challenges of studying the effect of person-centered care and not only in the measured effects, our content analysis approach resulted in: (1) an overview of definition and concepts; (2) an overview of interventions; (3) an overview of outcome measures and narrative description of effects; (4) a description of overall strengths and limitations of intervention studies as reported by the authors.

## Results

**Figure 1:**
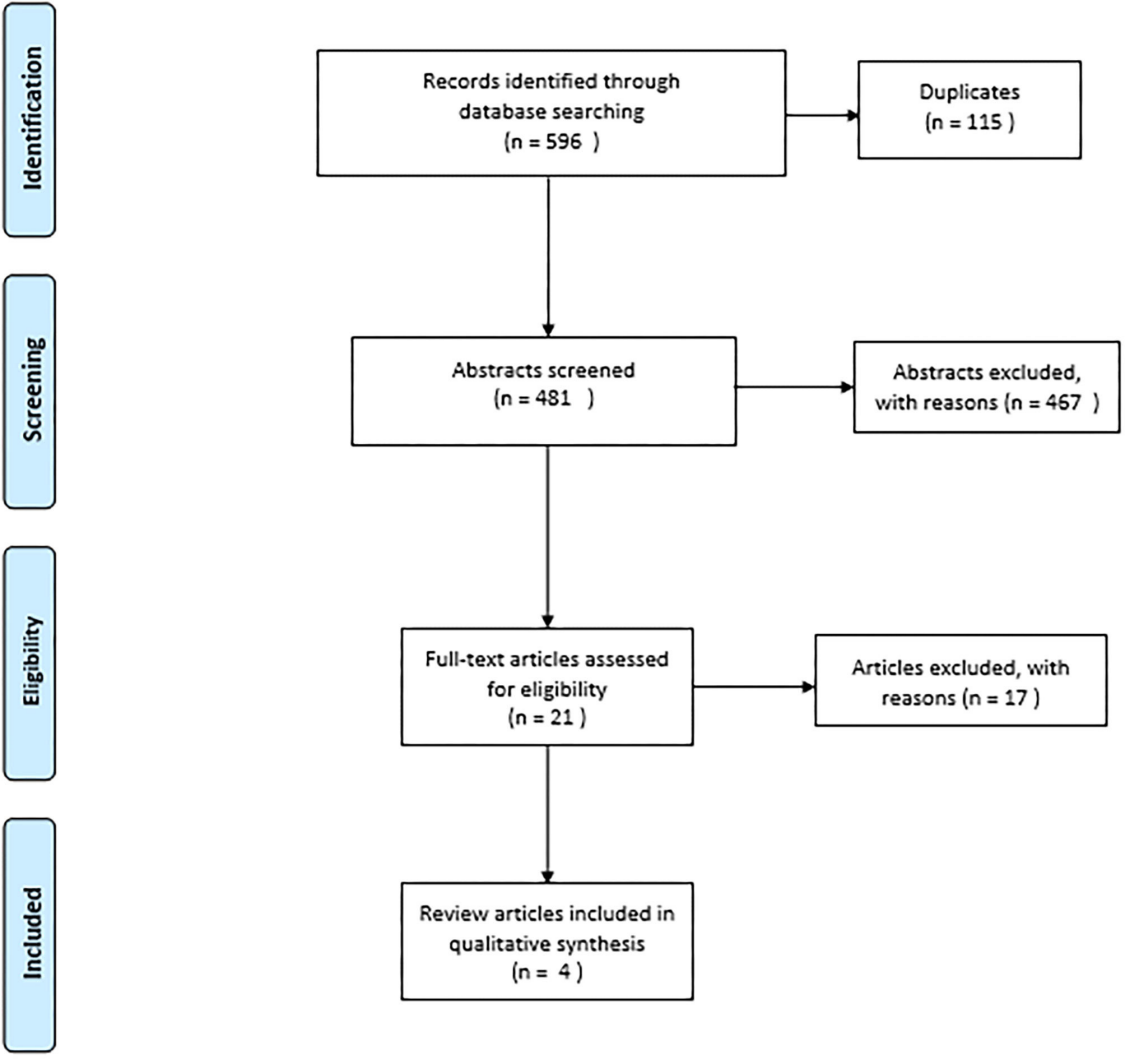
PRISMA flow diagram.

The search resulted in 596 hits. After duplicate removal, a total of 481 citations were reviewed for title and abstract screening (Figure 1). Most of these did not study persons-centered or patient-centered care as intervention and were excluded. Abstracts that described the effects of patient-centered medical homes and management of multimorbidity were considered eligible but were excluded after full text assessment as the intervention strategy also included management and care coordination to improve collaboration between healthcare providers. Four systematic reviews were finally included in this review (9, 14–16). The study characteristics of these reviews are presented in Table 1. The results of the content analysis are summarized in Table 2.

**Table 1:** Study characteristics.

**Author (year)**	**Type of review**	**Number of studies included**	**Setting**	**Countries**
Dwamena et al. (9)	Cochrane review of RCTs (update Lewin (8))	2000-2010: 30 1990-1999: 11 <1990: 2	Primary care: 35 Secondary or tertiary care: 13	USA: 15 UK: 9 Europe: 15 Other: 4
Rathert et al. (14)	Systematic review of empirical studies	2011-2012: 3 2000-2010: 32 1990-1994: 5	Not specified	USA: 14 UK: 5 Europe: 2 Other: 4 Unknown: 15
McMillan et al. (15)	Systematic review of RCTs	2011-2012: 4 2000-2010: 15 1990-1999: 7 <1990: 3	Primary care: 4 Hospital care: 2 Not specified: 23	Not specified
Park et al. (16)	Review of systematic reviews	2011-2017: 28 <2011: 0	Not specified	USA: 9 UK: 6 Australia: 6 Other:

**Table 2:** Overview of definitions, interventions, outcomes and effects, and strengths and limitations.

**References**	**Definitions and concepts**	**Interventions**	**Outcomes and effects**	**Strengths (S) and limitations (L)**
Dwamena et al. (9)	- Share control of consultations, decisions about interventions, or management of health problems, and/or - Focus on patient as person rather than disease	- Training for providers only - Training for providers combined with training or general educational materials for patients - Training for providers combined with condition-specific training or materials for providers - Training for providers combined with condition-specific materials or training for both providers and patients	- Positive on consultation process: 80% (28/35) - Positive on satisfaction: 46% (12/26) - Positive on behavior: 47% (8/17) - Positive on health status: 46% (12/26)	- S: focus on RCTs allowing meta-analysis - L: exclusion of non RCTs, heterogeneous and multiplicity of outcome measures
Rathert et al. (14)	- Respect for patient preferences - Information, education, communication - Coordination of care - Emotional support - Physical comfort - Involvement of family - Continuity & transition - Access to care	- Individualized treatment planning in collaboration with patients or training of practitioner - Information and communication processes - Focusing on all dimensions of quality of care - Focusing on other dimension of care	- Positive on satisfaction and patient well-being - In general mixed results on clinical and long-term outcomes - More positive outcomes	- L: inability to combine results of varied interventions, surveys, and outcome measures, difficulties in comparing interventions poorly described, small sample sizes in some studies
McMillan et al. (15)	- Holistic care - Individualized care - Respectful care - Empowering care.	- Complex interventions, e.g., Provision of tailored action plan, service referrals, follow-ups, and feedback - Simple intervention, one condition, e.g., Provision of medical record and treatment plan, lists of questions for patients - Training to providers to apply patient-centered care - Observation of interactions between patients and health providers	- Mixed findings, with improvements in some clinical indicators and negative impact on others - Positive on satisfaction when patients were engaged - Positive on quality of care, but little impact on clinical outcomes	- S: first systematic assessment of RCTs - L: variability in definitions and outcome measures, lack of detail about interventions, high risk of bias in most studies
Park et al. (16)	- Holistic approach to delivering respectful and individualized care, and - Offering choice through a therapeutic relationship where persons are empowered to be involved in health decisions	- Applied to patients, e.g., Physical support, education, training, and consulting, empowerment, emotional or environmental support - Applied to family members, e.g., Involvement in care, information sharing, shared decision making - Applied to healthcare providers, e.g., Education and training programs, coordination and continuity of care	- Positive on 75% (104/139) of outcomes - Neutral on 20% (28/139) of outcomes - Negative on 5% (7/139) of outcomes	- S: first review of systematic reviews on patient - and family-centered care-related interventions, comprehensive literature search of six electronic databases, followed by manual search - L: no consensus on definition, wide range of diverse subjects and interventions difficult to compare, limited quality of evidence from primary studies

### Study Characteristics

All reviews were narrative reviews, Dwamena et al. also included a meta-analysis of findings. Dwamena et al. and Park et al. performed a quality assessment of included studies using the Cochrane risk of bias tool and the AMSTAR tool, respectively. Park et al. included the other three reviews. According to the assessment of Park et al., Dwamena et al. met 9 out of 11 criteria of the AMSTAR tool, McMillan et al. 6 criteria, and Rathert et al. 5 criteria.

Most studies included were published between 2000 and 2010. Only two Randomized controlled trials were included in all reviews (17, 18). In Dwamena et al. 39 out of 43 studies were unique, in Rathert et al. 36 out of 40 studies, in McMillan et al. 24 out of 28 studies. Thirty-five (70%) studies included in Dwamena et al. were conducted in primary care, in the other reviews the setting was not specified, except in 6 studies in McMillan et al. Most studies were conducted in USA, followed by United Kingdom.

### Definition and Concepts

Dwamena et al. used two main features to define patient-centered care: (1) healthcare providers share control of consultations, decisions about interventions or the management of the health problems with patients, and/or (2) healthcare providers focus on the patient as a person, rather than solely on the disease, in consultations.

Rathert et al. used a conceptual framework based on Donabedian (19) considering patient-centered care as a process including: (1) respect for patient preferences; (2) information, education, communication; (3) coordination of care; (4) emotional support; (5) physical comfort; (6) involvement of family; (7) continuity & transition; and (8) access to care.

McMillan et al. adopted the model of Morgan and Yoder (20) on patient-centered care consisting of four features: (1) holistic care; (2) individualized care; (3) respectful care; and (4) empowering care.

Park et al. defined patient-centered care as “the holistic approach to delivering care that is respectful and individualized, allowing negotiation of care, and offering choice through a therapeutic relationship where persons are empowered to be involved in health decisions at whatever level is desired by that individual who is receiving the care,” also adopted from Morgan and Yoder (20).

### Interventions

Dwamena et al. classified the type of interventions into 4 categories, with the number of studies in brackets: (1) training for providers only (*n* = 23): (2) training for providers combined with training or general educational materials for patients (*n* = 7); (3) training for providers combined with condition-specific training or materials for providers (*n* = 7); and (4) training for providers combined with condition-specific materials or training for both providers and patients (*n* = 6).

Rathert et al. classified studies included in four categories: (1) patient preference studies (*n* = 19) examining individualized treatment planning in collaboration with patients or training of practitioner, particularly in consulting with patients; (2) studies on information and communication processes (*n* = 8) (21); (3) studies focusing on all Institute of Medicine (IOM) dimensions of quality of care (*n* = 9); (4) remaining dimension studies (*n* = 4). Only studies in category 1 included intervention studies.

McMillan et al. distinguished 4 categories of interventions: (1) complex interventions (*n* = 16): consisting of a number of components, i.e., provision of a tailored action plan, service referrals, follow-ups, and feedback; (2) simple intervention (*n* = 6): one environmental condition to facilitate a different style of interaction between patients and providers, i.e., provision of medical record and treatment plan for discussion, lists of questions for patients to ask providers; (3) training (*n* = 8): the delivery of skills or knowledge to providers to apply patient-centered care within their usual practice, i.e., communication workshops to develop listening skills, presentations on shared–decision making and cultural competency; (4) observational (*n* = 1): patients or health professionals view interactions between patients and health providers that either occur naturally or involve scripted vignettes and then rate the quality of the interactions or care provided to the patients. Three studies combined complex interventions with training.

Park et al. distinguished interventions applied to patients (*n* = 21), family members of patients (*n* = 9), and providers (*n* = 21). Reviews identifying interventions to patients were summarized in: (1) physical support (*n* = 16); (2) providing patients with information via tailored health education, training, and consulting (11); (3) patient empowerment (*n* = 13); (4) patient emotional support (*n* = 8); (5) patient involvement in care (*n* = 5); (6) assessment (*n* = 3); (7) environmental support (*n* = 2). Reviews addressing interventions to family members included interventions: (1) supporting the family as a unit of approach such as providing education sessions (*n* = 4); (2) involving family members in care activities (*n* = 6); (3) information sharing (*n* = 3); (4) shared decision making (*n* = 3); family support programs (*n* = 4). Interventions to healthcare providers were classified as: (1) education and training programs (*n* = 13); (2) coordination and continuity of care (*n* = 14); (3) other interventions such as teamwork and team building (*n* = 3), access to care (*n* = 5); and culture change (*n* = 2).

### Outcomes and Effects

Dwamena et al. classified the outcomes in 4 categories: (1) consultation process (*n* = 35); (2) satisfaction (*n* = 26); (3) healthcare behavior (*n* = 17); (4) health status (*n* = 26). All categories included outcomes with dichotomous variables and outcomes with continuous variables. The results of the meta-analysis were separately presented for these variables. The outcome measures used in all categories were unique, except the use of SF-12, SF-36 or the Spielberger (State-Trait Anxiety Inventory) in 3 to 4 studies for measuring the health status. The summary of the results showed that in 28 out of 35 studies (80%) the consultation process outcomes favored the intervention. Positive effects were found on a range of measures relating to patients' concerns and beliefs, communication on treatment options, levels of empathy, and patients' perception of providers' attentiveness to them and their concerns as well as their diseases. The percentage of studies favoring the intervention for the satisfaction outcomes, behavior outcomes, and health status outcomes was 46% (12/26), 47% (8/17), and 46% (12/26), respectively. Studies using complex interventions that focused on providers and patients with condition-specific materials generally showed benefit in satisfaction and health behavior, with mixed effects on health status.

Rathert et al. distinguished three categories: (1) patient satisfaction; (2) patient clinical outcomes; and (3) organizational outcomes. Outcomes measures were not specified for most studies, except for those using health outcomes (e.g., HbA1c, systolic blood pressure, BMI, or SF-12). The results were described narratively without quantitative analysis. The authors concluded that studies focusing on individualizing treatment plans for patients reported consistently greater satisfaction and patient well-being. The studies found mixed results on clinical and long-term outcomes, with some finding relationships with outcomes (hospital readmission, complications) and others finding no relationship. Studies focusing on all dimensions of quality of care seemed to be more consistent in finding positive outcomes. Emotional support may encourage patient activation and adherence with treatment plans, which lead to better patient outcomes.

McMillan et al. also used three categories: (1) patient satisfaction (*n* = 14); (2) perceived quality of care (*n* = 11); and (3) health outcomes (*n* = 21), which were further categorized in clinical outcomes (*n* = 6), functional outcomes (*n* = 12), personal outcomes (*n* = 14); and system outcomes (*n* = 8). Except for the PACIC (Patient Assessment of Chronic Illness Care) and a few clinical outcomes (e.g., BMI, blood pressure, HbA1c), all outcome measures were unique. The results were described narratively without quantitative analysis, similar as Rathert et al. The authors concluded that increases in satisfaction were found when patients were prompted to actively engage in the consultation rather than simply being given information. Training of providers contributed to increased quality of care as perceived by both providers and patients, but had little impact on clinical outcomes. Functional outcomes improved but not on the long term. Simple interventions, such as provision of a treatment plan or lists of questions for patients, designed to increase empowerment and active engagement during the medical decision-making process had a positive impact. In contrast, complex interventions resulted in mixed findings, with improvements in some clinical indicators and a negative impact on others.

Park et al. reported the outcomes to patient- and family-centered care interventions applied to patients, family members, or health-care providers without further classification and without specifying the instruments used to measure the outcomes. As review of reviews, it included all above mentioned outcomes, as well as dimensions of quality of care, such as access, and economic outcomes, in total 139 (mean of 5 per study). The findings were summarized per review and per outcome using a three point scale (+, ±, and –). Positive results were reported on 75% (104/139) of the outcomes, 20% (28/139) were neutral, and 5% (7/139) were negative.

The authors concluded that interventions targeting patients were improving knowledge about their health, increasing skills to manage self-care behaviors, enhancing satisfaction, increasing quality of life, and reducing admissions, readmissions, and length of the hospital stay. Interventions targeting family members were reducing the intensity of stress, anxiety, depression, and increasing the satisfaction and relationship with health-care providers. Interventions targeting health-care providers could improve job satisfaction and confidence, quality of care, and reduce stress and burnout.

### Overall Strengths and Limitations

Dwamena et al. noted that the strength of the review was the focus on Randomized controlled trials allowing meta-analysis, but this was a limitation as well as it excluded studies with other designs, particularly those undertaken in the context of descriptive healthcare system quality improvement. Heterogeneous outcome measures limited the pooling of results. The multiplicity of outcome measures resulted in participants scoring positively on some and negatively on other skills, leaving an unclear pattern of overall patient-centeredness.

Rathert et al. did not explicitly reported strengths of the study and mentioned the following limitations: inability to combine the results of varied interventions, surveys, and outcome measures; difficulties in comparing different interventions when they are poorly described; and small sample sizes in some of the studies, limiting the generalizability to other populations.

McMillan et al. noted that their review represented the first systematic assessment of Randomized controlled trials supporting the efficacy of interventions promoting patient-centered care, specifically for people with chronic illnesses. Limitations are related to the variability in the definition of patient centered care, the outcome measures used, the lack of detail about the actual interventions in the studies, and high risk of bias in most studies, reflecting the complexity of this field of research.

Park et al. noted that their study was the first review of systematic reviews of research evidence of patient- and family-centered care-related interventions in healthcare. Another strength was that they performed a comprehensive literature search of six electronic databases, followed by a manual search of the reference lists of selected relevant reviews. In addition, study selection, data extraction, and quality appraisal were carefully and specifically performed. Limitations were that there was no consensus regarding the definition of patient- and family centered care in the identified reviews involving diverse participants, settings, purposes, and strategies, which made it difficult to pool the findings of the reviews. The findings covered a wide range of diverse subjects, which led to difficulties in synthesizing clear results. The interventions might have focused on only a few aspects of patient- and family-centered care that made it difficult to assess and compare among the various interventions. The results were also constrained by the quality of evidence from the primary studies.

## Discussion

In this scoping review, we presented and analyzed the results of four systematic reviews studying the effects of interventions to deliver person-centered or patient-centered care. In total, the reviews included 145 unique studies, most of these were Randomized controlled trials. Although the most recent review (16) included the other reviews, the lack in overlap of included studies was surprising. Different definitions and concepts were used resulting in different search strategies and selection criteria. Two reviews used the model of Morgan and Yoder but with different operationalizations (14, 16). Within the reviews, there was also a large variety of interventions. Two reviews generally distinguished interventions for providers and for patients, but these were differently classified (9, 16). One review specified family-oriented interventions (16). In reporting the effects, the reviews classified the outcomes differently, whereas patient satisfaction was specified by all of them. Two reviews explicitly distinguished process outcomes and health or clinical outcomes (9, 14). The differences in definitions, interventions, and outcomes of studies included was acknowledged as a limitation by the authors of all reviews. As a result, combining or pooling the effects of studies was difficult and subject to bias.

Person-centered care is a concept that seems obvious and understandable in real life but is complex to operationalize in research (22). This also applies to other core values of general practice, such as continuity and integrated care (23, 24). Researchers face the dilemma to study the effect of a single intervention, such as a tool or treatment plan, and to focus on one disease or condition, or a complex of interventions to optimize the effect and its generalizability, which is more difficult to analyze due to its different components. This confirms the need to use mixed methods in general practice research (25).

All reviews showed positive effects of interventions promoting person-centered care. The effects on process outcomes, such as the consultation process, were convincing but effects on patient satisfaction and health outcomes were mixed. Process outcomes are more sensitive to differences in quality of care, whereas patient relevant outcomes are of greater intrinsic interest and can reflect all aspects of care (26). Empirical studies that investigated the relationship between patient-centered consultation and patient outcomes in primary care, however, often have shortcomings in internal and external validity (27, 28). If efforts are made to standardize data collection and to adjust to case mix, patient outcome measures could be improved. Nevertheless, lack of effects on patient outcomes can also be due to small sample sizes and limited time to follow-up. Therefore, focusing on the process maybe a pragmatic choice assuming that effects on patient outcomes will come later if process improvements are sustainable.

We were also interested in the effects of patient-centered care on costs. However, only three studies included in one of the reviews studied the economic effects (16). Although positive effects were reported, the generalizability is limited as two studies focused on children and their families and a third study examined care for patients after hip fracture. The assessment of the effects of patient-centered medical homes in the United States also included economic outcomes showing small but significant effects compared to standard care (29). The interventions also included changes in management and coordination of care, which might dilute the effects of patient-centered care. Another review assessing the effects of personalized care planning for adults with long-term health conditions showed limited and uncertain evidence on the relative cost effectiveness of this approach (30). The focus on management was the reason that we excluded these studies in our review.

In sum, our scoping review showed that effects on patient outcomes are difficult to assess due to heterogeneity of definitions, interventions, and outcomes. A limitation of this study is that we only included four systematic reviews. We think, however, that a more sensitive search resulting in inclusion of more reviews would not change the results. Moreover, all authors of the reviews included came to the same conclusions. Another limitation is that the two reviews that did a quality assessment did not exclude studies of low quality. In particular, contamination could be a problem as some interventions cannot be concealed from patients or providers. Third, the review of Park et al. included the other reviews, which might raise the question why we still included these reviews. In this scoping review, however, we were particularly interested in the research methods and not only in the effects of person-centered care. Inclusion of the earlier reviews enriched our review as they used different definitions, classification of interventions, and outcomes. Final limitation is that all reviews did not focus exclusively on general practice. The setting of many studies included in the reviews was not reported. As most of the studies were conducted in the United States, we need to be cautious to extrapolate the results to countries with a different healthcare system.

As there is broad consensus on person-centered care being a core value of general practice, one might argue whether there is still a need for evidence of positive effects. Would a lack of research evidence change our opinion that person-centered is crucial for primary care? We do not think it would. For adding value to the body of knowledge, an implementation or complexity science approach could be used for spreading and scaling up person-centered care (31). The first takes a structured and stepwise approach to developing, replicating, and evaluating an intervention that have shown effectiveness, and the latter encourages a flexible and adaptive approach to change in a dynamic, self-organizing system assuming uncertainty and unpredictability. Both use multiple qualitative and quantitative methods for analyzing improvement.

Sharing the goal of improving the care of individual patients, “individual point-of-care trials” integrating clinical research and patient-centered care could also be considered (32). These build on the framework of N-of-1 studies. As many patients are similar but all patients are different, research in general practice could be personalized by defining therapeutic goals, interventions, and outcome variables based on individual preferences, goals, and values and not only on clinical and biological characteristics. In the context of education and training observational data from real practice and patient satisfaction surveys could be used to support quality improvement. Thus, integrating research, education, and practice could strengthen the profession, building on the fundament of shared core values.

## Author Contributions

JSB and TW conceived the study and developed the methodology of the study. JSB and EWB selected and assessed the articles. JSB, EWB, and TW interpreted the articles. JSB wrote the first draft of the manuscript. EWB and TW revised the first draft of the manuscript. All authors have read and approved the final version of the manuscript.

## Conflict of Interest

The authors declare that the research was conducted in the absence of any commercial or financial relationships that could be construed as a potential conflict of interest.
