# Supplementary material for: Challenges of Research on Person-Centered Care in General Practice: A Scoping Review
Source: Front Med (Lausanne). 2021 Jun 24;8:669491. doi: 10.3389/fmed.2021.669491 (PMC8264253; doi:10.3389/fmed.2021.669491)
Supplement: Supplementary file 1 [file Data_Sheet_1.DOCX]

# Search Strategies Scoping Review Effects of Person-Centered Care

Last search date (all databases): 2021-02-01

##### **Medline (PubMed)**

("General Practice"[mh] OR "Primary Health Care"[mh] OR "Physicians, Primary Care"[mh] OR "Physicians, Family"[mh] OR "General Practitioners"[mh] OR General-practi*[tiab] OR primary-health-care[tiab] OR primary-healthcare[tiab] OR primary-care[tiab] OR family-doctor*[tiab] OR family-physician*[tiab] OR family-practi*[tiab] OR family-medicine[tiab])

AND

("Patient-Centered Care"[mh] OR "Decision Making, Shared"[Mh] OR Patient-focused[tiab] OR Patient-centered*[tiab] OR Patient-centred*[tiab] OR person-centered*[tiab] OR person-centred*[tiab] OR patient-tailored[tiab] OR Individualized-care[tiab] OR personalized-care[tiab] OR personalised-care[tiab] OR shared-decision[tiab])

AND

("Patient Outcome Assessment"[Mh] OR patient-reported-outcom*[tiab] OR patient-outcom*[tiab] OR PROM[tiab] OR PROMs[tiab] OR outcome*[ti] OR effectiv*[ti] OR (quality[ti] AND care[ti]) OR

"Quality of Life"[Mh] OR quality-of-life[tiab] OR qol[tiab] OR hrql[tiab] OR hrqol[tiab] OR life-quality[tiab] OR "Patient Satisfaction"[Mh] OR patient-satisf*[tiab] OR patients-satisf*[tiab] OR patient’s-satisf*[tiab] OR patients’-satisf*[tiab] OR satisfaction[ti] OR patient-preferenc*[tiab] OR patient’s-preferenc*[tiab] OR patients-preferenc*[tiab] OR patient-experienc*[tiab] OR patient-perspectiv*[tiab] OR patient’s-perspectiv*[tiab] OR patients-perspectiv*[tiab] OR patient-particip*[tiab] OR patient-empower*[tiab] OR patient-engag*[tiab] OR patient-involv*[tiab] OR patients-involv*[tiab] OR patient’s-need*[tiab] OR patients-need*[tiab] OR patient-need*[tiab] OR (patient*[ti] AND (preferenc*[ti] OR perspectiv*[ti] OR experience*[ti] OR empower*[ti] OR involv*[ti] OR engag*[ti])) OR "economics"[Subheading] OR "Costs and Cost Analysis"[Mh] OR cost*[ti] OR econom*[ti] OR cost-effect*[tiab] OR cost-benefit[tiab] OR cost-analys*[tiab])

**AND**

**(systematic review[pt] OR meta-analysis[pt] OR meta-anal*[tiab] OR metaanal*[tiab] OR quantitative-review[tiab] OR systematic-review[tiab] OR methodologic-review[tiab] OR systematic[sb])**

**395 hits**

##### **Embase (Elsevier)**

('general practice'/exp OR 'general practitioner'/exp OR 'primary health care'/exp OR 'family medicine'/exp OR General-practi*:ab,ti OR primary-health-care:ab,ti OR primary-healthcare:ab,ti OR primary-care:ab,ti OR family-doctor*:ab,ti OR family-physician*:ab,ti OR family-practi*:ab,ti OR family-medicine:ab,ti)

AND

('patient centered outcomes research'/exp OR 'patient centeredness'/exp OR 'patient centered outcome'/exp OR 'patient centered communication'/exp OR 'shared decision making'/exp OR 'personalized medicine'/exp/mj OR Patient-focused:ab,ti OR Patient-centered*:ab,ti OR Patient-centred*:ab,ti OR person-centered*:ab,ti OR person-centred*:ab,ti OR patient-tailored:ab,ti OR Individualized-care:ab,ti OR personalized-care:ab,ti OR personalised-care:ab,ti OR shared-decision:ab,ti)

AND

('outcome assessment'/de OR 'patient-reported outcome'/exp OR patient-reported-outcom*:ab,ti OR patient-outcom*:ab,ti OR PROM:ab,ti OR PROMs:ab,ti OR outcome*:ti OR effectiv*:ti OR (quality:ti AND care:ti) OR 'quality of life'/exp OR quality-of-life:ab,ti OR qol:ab,ti OR hrql:ab,ti OR hrqol:ab,ti OR life-quality:ab,ti OR 'patient satisfaction'/exp OR patient-satisf*:ab,ti OR patients-satisf*:ab,ti OR patient-s-satisf*:ab,ti OR patients--satisf*:ab,ti OR satisfaction:ti OR patient-preferenc*:ab,ti OR patient-s-preferenc*:ab,ti OR patients-preferenc*:ab,ti OR patient-experienc*:ab,ti OR patient-perspectiv*:ab,ti OR patient-s-perspectiv*:ab,ti OR patients-perspectiv*:ab,ti OR patient-particip*:ab,ti OR patient-empower*:ab,ti OR patient-engag*:ab,ti OR patient-involv*:ab,ti OR patients-involv*:ab,ti OR patient-s-need*:ab,ti OR patients-need*:ab,ti OR patient-need*:ab,ti OR (patient*:ti AND (preferenc*:ti OR perspectiv*:ti OR experience*:ti OR empower*:ti OR involv*:ti OR engag*:ti)) OR 'cost'/exp OR 'economic evaluation'/exp OR cost*:ti OR econom*:ti OR cost-effect*:ab,ti OR cost-benefit:ab,ti OR cost-analys*:ab,ti)

AND ('systematic review'/exp or 'meta analysis'/exp OR systematic-review:ab,ti OR meta-anal*:ab,ti OR metaanal*:ab,ti OR quantitative-review:ab,ti OR methodologic-review:ab,ti ) NOT 'conference abstract'/it

**169 hits**

##### **PsycInfo (EBSCOhost)**

( DE "General Practitioners" OR DE "Primary Health Care" OR DE "Family Medicine" OR TI (“General practi*” OR “primary health care” OR “primary healthcare” OR “primary care” OR “family doctor*” OR “family physician*” OR “family practi*” OR “family medicine”) OR AB (“General practi*” OR “primary health care” OR “primary healthcare” OR “primary care” OR “family doctor*” OR “family physician*” OR “family practi*” OR “family medicine”)) AND ( DE "Patient Centered Care" OR TI (“patient focused” OR “patient centered*” OR “patient centred*” OR “person centered*” OR “person centred*” OR “patient tailored” OR “individualized-care” OR “personalized care” OR “personalised care” OR “shared-decision”) OR AB (“patient focused” OR “patient centered*” OR “patient centred*” OR “person centered*” OR “person centred*” OR “patient tailored” OR “individualized-care” OR “personalized care” OR “personalised care” OR “shared-decision”) ) AND ((DE "Patient Reported Outcome Measures" OR TI (“patient reported outcom*” OR “patient outcome*” OR “PROM” OR “PROMs” OR “outcom*” OR “effectiv*” OR (“quality” AND “care”)) OR AB (“patient reported outcom*” OR “patient outcome*” OR “PROM” OR “PROMs”) OR DE “Quality of Life” OR DE “Health Related Quality of Life” OR TI (“quality of life” OR “qol” OR “hrql” OR “hrqol” OR “life quality”) OR AB (“quality of life” OR “qol” OR “hrql” OR “hrqol” OR “life quality”) OR DE “Client Satisfaction” OR TI (“patient satisf*” OR “patients satisf*” OR “patient’s satisf*” OR satisfaction OR “patient preferenc*” OR “patient’s preferenc*” OR “patients preferenc*” OR “patient experienc*” OR “patient perspectiv*” OR ”patient’s perspectiv*” OR “patients perspectiv*” OR “patient particip*” OR “patient-empower*” OR “patient engag*” OR “patient involv*” OR “patients involv*” OR “patient need*” OR “patient’s need*” OR “patients need*” OR (“patient*” AND (“preferenc*” OR “perspectiv*” OR “experience*” OR “empower*” OR “involve*” OR “engag*”)) OR AB (“patient satisf*” OR “patients satisf*” OR “patient’s satisf*” OR “patient preferenc*” OR “patient’s preferenc*” OR “patients preferenc*” OR “patient experience*” OR “patient perspective*” OR ”patient’s perspective*” OR “patients perspective*” OR “patient particip*” OR “patient-empower*” OR “patient engag*” OR “patient involve*” OR “patients involve*” OR “patient need*” OR “patient’s need*” OR “patients need*”) OR DE “Costs and Cost Analysis” OR DE “Health Care Costs” OR DE “Health Care economics” OR TI (“cost*” OR “econom*”) OR AB (“cost effect*” OR “cost benefit” OR “cost analys*”) )) AND ( MR (“systematic review”) OR TI (“systematic review*” OR “meta-anal*” OR “metaanal*” OR “quantitative review” OR “methodologic review” OR “integrative review*”) OR AB (“systematic review*” OR “meta-anal*” OR “metaanal*” OR “quantitative review” OR “methodologic review” OR “integrative review*”))

**32 hits**

395 + 169 + 32 = 596 hits – 115 duplicates --> 481 **unique hits**
